# Role for the thromboxane A_2_ receptor β-isoform in the pathogenesis of intrauterine growth restriction

**DOI:** 10.1038/srep28811

**Published:** 2016-07-01

**Authors:** Katie L. Powell, Veronica Stevens, Dannielle H. Upton, Sharon A. McCracken, Ann M. Simpson, Yan Cheng, Vitomir Tasevski, Jonathan M. Morris, Anthony W. Ashton

**Affiliations:** 1 Division of Perinatal Research, Kolling Institute, Northern Sydney Local Health District, St Leonards, NSW, 2065, Australia; 2 Sydney Medical School Northern, University of Sydney, NSW, 2006, Australia; 3 Pathology North, NSW Health Pathology, Royal North Shore Hospital, St Leonards, NSW, 2065, Australia; 4 School of Life Sciences, University of Technology Sydney, Ultimo, NSW, 2007, Australia; 5 Centre for Health Technologies, University of Technology Sydney, Ultimo, NSW, 2007, Australia; 6 Institute for Translational Medicine and Therapeutics, University of Pennsylvania School of Medicine, Philadelphia, Pennsylvania, 19104, USA

## Abstract

Intrauterine growth restriction (IUGR) is a pathology of pregnancy that results in failure of the fetus to reach its genetically determined growth potential. In developed nations the most common cause of IUGR is impaired placentation resulting from poor trophoblast function, which reduces blood flow to the fetoplacental unit, promotes hypoxia and enhances production of bioactive lipids (TXA_2_ and isoprostanes) which act through the thromboxane receptor (TP). TP activation has been implicated as a pathogenic factor in pregnancy complications, including IUGR; however, the role of TP isoforms during pregnancy is poorly defined. We have determined that expression of the human-specific isoform of TP (TPβ) is increased in placentae from IUGR pregnancies, compared to healthy pregnancies. Overexpression of TPα enhanced trophoblast proliferation and syncytialisation. Conversely, TPβ attenuated these functions and inhibited migration. Expression of the TPβ transgene in mice resulted in growth restricted pups and placentae with poor syncytialisation and diminished growth characteristics. Together our data indicate that expression of TPα mediates normal placentation; however, TPβ impairs placentation, and promotes the development of IUGR, and represents an underappreciated pathogenic factor in humans.

Intrauterine growth restriction (IUGR) is defined as failure of the fetus to reach its genetically pre-determined growth potential^1^,^2^. IUGR can be diagnosed *in utero* by estimated fetal weight for gestational age less than the 10^th^ percentile, accompanied by Doppler ultrasound detecting decreased, absent or reversed end diastolic flow in the umbilical arteries^1^,^3^. IUGR is a leading cause of fetal mortality and morbidity, second only to pre-term delivery^1^,^4^,^5^,^6^; however, IUGR offspring that survive the perinatal period are at increased risk of cardiovascular disease and renal complications in adulthood^7^. Currently no biomarkers exist that predict pregnancies at risk of developing IUGR nor are there effective preventative treatments. In the absence of effective prophylaxis or treatment the growth restricted fetus is delivered early, often pre-term, which contributes further to the risk of adverse neonatal outcomes^8^.

IUGR can be classified as either symmetrical or asymmetrical. Symmetrical IUGR results from maternal factors (such as malnutrition and alcohol consumption) and congenital infections. Conversely, asymmetrical growth primarily arises from the presence of a placental pathology^2^,^9^. Placentae of growth restricted pregnancies are smaller in weight, have reduced number and diameter of villi and a reduction in the number and area of capillaries within the villi indicating impaired vascularisation and branching angiogenesis^10^,^11^,^12^. They also exhibit greater rates of infarction, fetal vessel thrombosis and chronic villitis than the placentae of normal pregnancies^13^. These placental pathologies are precipitated by morphological and biochemical abnormalities at the cellular level. IUGR placentae display increased apoptosis, reduced cytotrophoblast proliferation, diminished syncytiotrophoblast area with an increased number of nuclei present indicative of compromised syncytialisation^10^,^12^,^14^. Insufficient trophoblast invasion, with failure to remodel spiral arteries, is also present^15^,^16^. The abnormal trophoblast function and reduced placental vascularisation shift the physiologic equilibrium in the placenta promoting placental hypoxia and ultimately oxidative stress^10^.

Reactive oxygen species (ROS), produced in response to oxidative stress, initiate damage to a variety of intracellular molecules including DNA, protein and membrane lipids. Peroxidation of the membrane lipid arachidonic acid results in the formation of F-series isoprostanes, which are markers of oxidative stress^17^,^18^. However, isoprostanes are also biologically active and exert their effects through activation of the thromboxane (TX)A_2_ receptor (TP)^17^,^19^. *Bone fide* TXA_2_ generation is also perturbed in IUGR with the TXA_2_:prostacyclin ratio increased in maternal serum and perfusates of the placental circulation from IUGR pregnancies compared to those with “normal” outcomes^20^,^21^. TXA_2_ overproduction by placentae and platelets is prevalent in human pregnancies complicated by cigarette smoking^22^, diabetes mellitus^23^ and alcohol consumption^24^ which are associated with IUGR. Furthermore, infusion of TXA_2_ analogues (such as U46619 and STA_2_) into rodents produces a IUGR-like syndrome characterised by a 10–15% reduction in pup weight, which persisted until 28 and 77 days post-partum for male and female offspring, respectively^25^,^26^,^27^. The pups from these pregnancies exhibit a pattern of growth restriction characteristic of asymmetrical IUGR with reduced body/liver weight with sparing of brain changes in weight or energy metabolites^25^,^28^,^29^. Moreover, TP antagonists block the vasoconstriction and normalised pup and placental weight in rodent models associated with IUGR^30^,^31^, suggesting a strong causal role for TXA_2_ in disease.

The role of the TP is well established in a number of diseases; however, very little information exists on the role of the specific isoforms during pregnancy. The G-protein coupled TP has two isoforms in humans, TPα and TPβ, derived from alternate splicing of a single gene^32^,^33^. Both isoforms are identical through the first 328 amino acids but differ in the length of the cytoplasmic C-terminus (with the tails 15 and 79 amino acids for TPα and TPβ, respectively). The cytoplasmic tail is a poor discriminator of G-protein coupling^34^,^35^ promoting complacency over the differential role of these two isoforms in human disease. Our study sought to identify the complement of TP isoforms expressed in placentae from normal, healthy and IUGR affected pregnancies and to understand the differential regulation of trophoblast function by TPα and TPβ. We have identified enhanced TPβ protein expression in placentae from IUGR pregnancies compared to those from normal pregnancies. Moreover, we demonstrate that enhanced TPβ expression impairs trophoblast function promoting placental insufficiency that is characteristic of IUGR. Pup weight and placental density in the TPβ-transgenic mice are significantly reduced and trophoblasts in these placentae exhibit impaired proliferation and syncytialisation consistent with IUGR in humans. In conclusion, we propose that dysregulation of TP isoform expression in the placenta is a significant cause of spontaneous IUGR in human pregnancy, which is amenable to targeted therapy to prevent both the short and long term mortality and morbidity associated with this condition.

## Results

### TPβ expression is increased in the syncytial layer of placentae from growth restricted pregnancies

**Figure 1:**
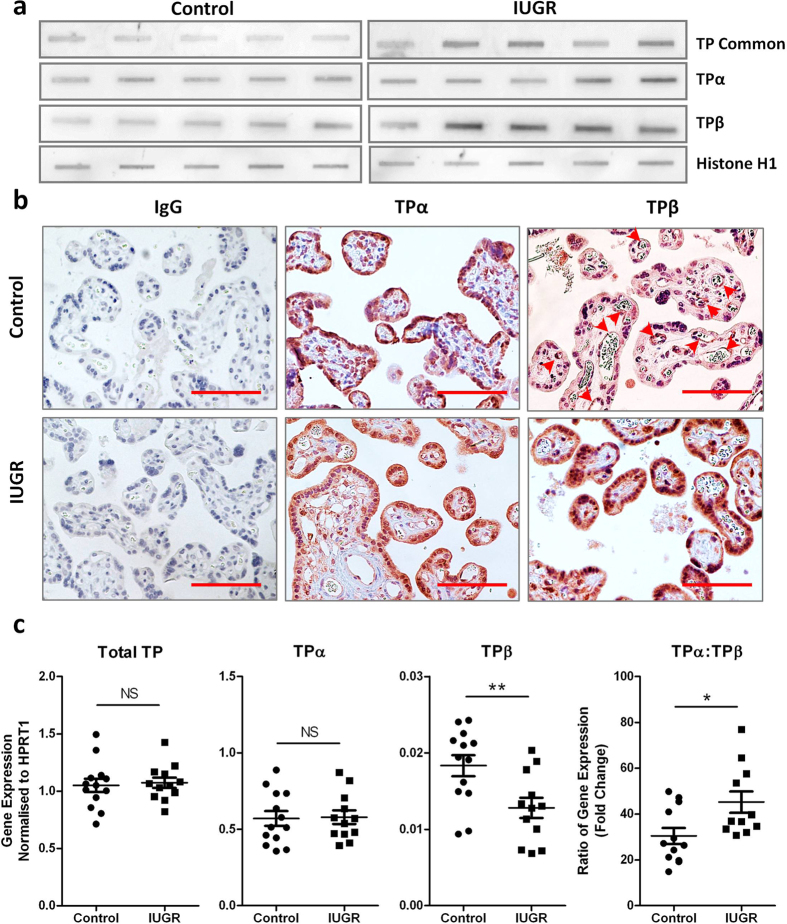
TPβ is highly expressed in IUGR placentae but is absent from healthy placentae. (**a**) Slot blots on lysates from normal, healthy placentae and placentae from pregnancies complicated by IUGR were probed with antibodies to identify total TP expression and that of the two isoforms (TPα and TPβ) in addition to a loading control (Histone H1) (*n* = 17 control and 15 IUGR placentae). (**b**) IHC on paraffin-embedded sections from normal, healthy placentae and placentae from pregnancies complicated by IUGR were probed with antibodies to TPα and TPβ. Sections were imaged at 200x magnification, scale bar: 100 μm; arrows indicate staining in vascular endothelium (*n* = 53 control and 22 IUGR placentae). (**c**) Quantitative gene expression of total TP, TPα and TPβ (and change in the ratio of TPα:TPβ) relative to HPRT1 expression was assessed in the same placental samples analysed in (**a**) (*n* = 13 control and 12 IUGR placentae). Data represent individual samples (mean ± SEM); Independent samples T-test, NS: not significant, *P < 0.05, **P < 0.01.

The distribution and expression of TP isoforms in placental tissue, and whether this changes during IUGR, has not been documented. We assessed TP isoform expression in third trimester placental tissue from “normal” uncomplicated pregnancies and those affected by IUGR (Table 1). As expected, the birth weight of babies from IUGR pregnancies was significantly lighter (*P* = 0.004) compared with those from control gestationally matched pregnancies (Table 1). Immunoblotting of placental protein extracts revealed a 2.8 fold increase in overall TP expression in IUGR placentae (*P* < 0.005) (Fig. 1a). This was associated with increased expression of both TPα (1.6 fold; *P* = 0.017) and TPβ (2.6 fold; *P* < 0.005) (Fig. 1a) after normalisation to the loading control. When examined by IHC, TPα expression was ubiquitous in control placentae (including the syncytiotrophoblast layer, endothelial cells and villous stroma); however, TPβ expression was essentially absent, with any staining primarily restricted to the endothelium of villous blood vessels (Fig. 1b, arrows). Conversely, TPβ expression was markedly increased in the syncytiotrophoblast layer in IUGR tissues while TPα expression was not different (Fig. 1b). TP isoforms are regulated by alternative splicing. Transcript levels for Total TP and TPα did not change in IUGR; however significantly lower expression of TPβ was evident in IUGR compared to control placental tissues (Fig. 1c). This change in TPβ gene expression also contributed to a change in the ratio of TPα:TPβ (Fig. 1c). These data suggest that neither transcription nor alternate splicing were responsible for the enhanced TPβ protein expression and that protein stabilisation is the most likely explanation.

### TP isoforms differentially regulate trophoblast proliferation

**Figure 2:**
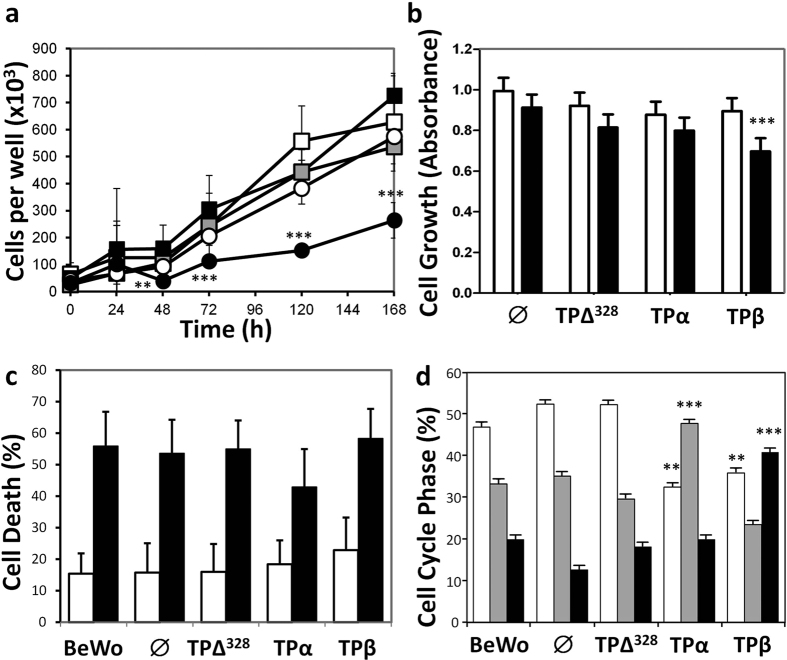
Differential effects of TP isoforms on trophoblast cell cycle. (**a**) Proliferation of BeWo cells (□) transfected with empty vector-∅ (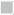), TPΔ^328^ (O), TPα (■) and TPβ (●) was monitored over 168 hours. (**b**) Proliferation of JEG-3 cells expressing similar constructs, stimulated with vehicle (□) or 100 nM I-BOP (■) was documented over the same period. (**c**) Apoptosis of BeWo cells under basal (□) and serum-deprivation (■) was assessed after 24 hours using flow cytometry. (**d**) Distribution of transfected BeWo lines in phases of the cell cycle (G0/G1 □, S 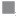, G2/M ■) was assessed also using flow cytometry. The data are represented as mean ± SEM and are representative of 3 independent experiments. One-way ANOVA and Independent samples T-test, *P < 0.05, **P < 0.01, ***P < 0.005.

To explore the individual role of TP in placentation we characterised the effects of TPα and TPβ in the choriocarcinoma cell lines, BeWo and JEG-3, commonly used as *in vitro* models of placental development. Both BeWo and JEG-3 cells constitutively expressed low levels of TPα but no TPβ (Supplementary Fig. S1). Transfection and stable selection of TPα, TPβ and the “tailless” deletion mutant (TPΔ^328^) led to a 5–10 fold increase in receptor expression at the protein level compared to empty vector (∅) transfected cells (Supplementary Fig. S1). Overexpression of TPΔ^328^ or TPα conferred no growth advantage compared to ∅ transfected cells or untransfected BeWo (Fig. 2a) or JEG-3 (Fig. 2b) cells. However, activation of TPβ ablated proliferation in BeWo cells (*P* = 0.014) and reduced growth of JEG-3 cells by 30% (*P* = 0.001), (Fig. 2a,b, respectively). Thus, TP isoforms show specific and opposite regulation of trophoblast function.

**Figure 3:**
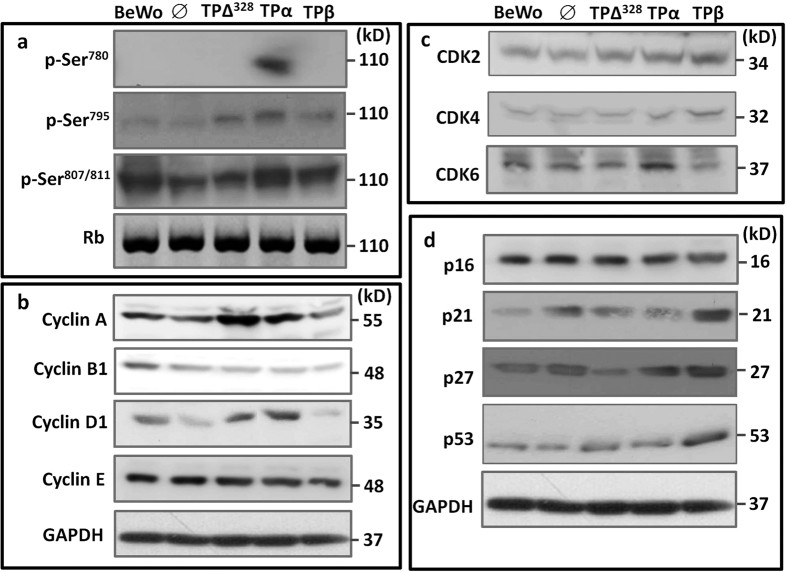
TP isoforms differentially regulate the molecular machinery controlling cell cycle progression. Immunoblotting of BeWo cells transfected with empty vector (∅), TPΔ^328^, TPα and TPβ was used to examine pRb phosphorylation (**a**), Cyclins (**b**), Cyclin-dependent kinases (CDK) (**c**) and tumour suppressor proteins (**d**). Non-phosphorylated Rb was used as the loading control in panel (**a**) and GAPDH was used as the loading control in panels (**b-d**). Blots are representative of 4 individual experiments. Densitometry was used to quantify changes in protein expression and activation status.

Proliferation is a balance between cell division and cell death. Examination of apoptosis by flow cytometry, either at baseline or under serum-starvation conditions, did not show differences between any of the TP transfected cell lines and the ∅ control (Fig. 2c). Therefore the effects of TPβ on trophoblast growth must be related to cell cycle progression. Examination of randomly cycling cells indicated expression of TPΔ^328^ had no effect on BeWo cell cycle progression compared to ∅ transfected or untransfected BeWo cells (Fig. 2d). TPα over expression elevated the number of cells in S-phase (Fig. 2d) suggesting enhanced G_0_/G_1_ exit which was confirmed by reduced ability to arrest cells with L-Mimosine (Supplementary Fig. S2) and enhanced S-phase entry within 24 hours of withdrawal of L-Mimosine (Supplementary Fig. S2). This was accompanied by enhanced phosphorylation of Ser^780^ and Ser^795^ in Rb in TPα expressing cells (Fig. 3a), the product of enhanced expression of Cyclin A (3.2 fold), D1 (2.6 fold) and CDK6 (4.1 fold) (Fig. 3b,c). Conversely, TPβ activation promoted accumulation of cells in the G2/M phase (Fig. 2d) suggesting the reduced proliferation was due to an inability to complete cell division. This phase of the cell cycle is driven by Cyclin B-CKD1 complexes, which appear to be normally expressed in TPβ-BeWo cells (Fig. 3b). However, expression of the cell cycle inhibitor proteins p21 (5.2 fold), p27 (3.9 fold) and p53 (2.6 fold) were all increased with TPβ overexpression compared to TPα, TPΔ^328^, ∅ and untransfected BeWo cells (Fig. 3d; *P* < 0.05). Therefore the cells were unable to progress through the G2/M phase causing inhibition of TPβ cell proliferation (Fig. 2a).

### TPβ expression reduces BeWo syncytialisation

**Figure 4:**
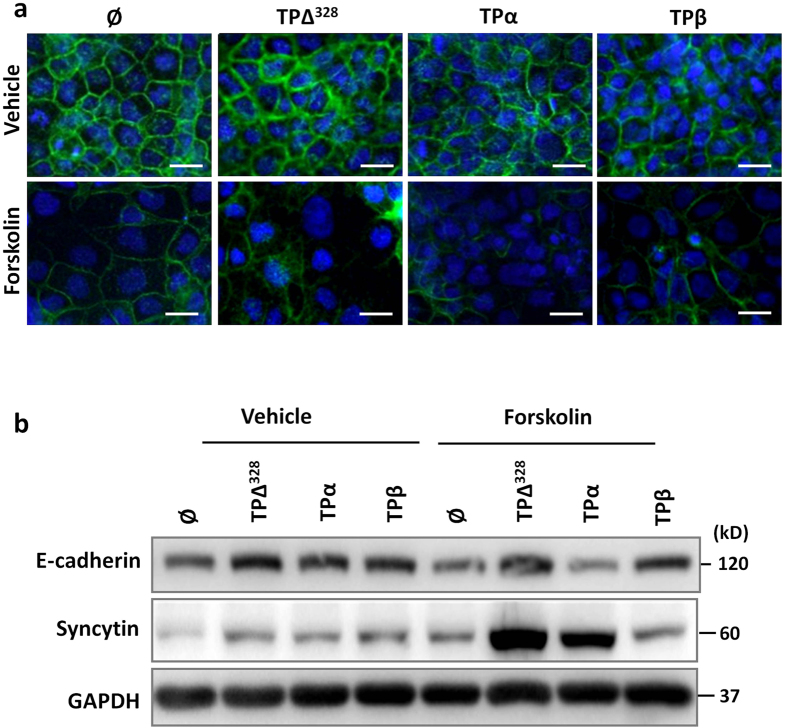
TPβ slows and TPα enhances the rate of syncytialisation of trophoblast cells. (**a**) Transfected BeWo cells were incubated for 72 hours with 200 nM I-BOP (vehicle) or 200 nM I-BOP and 100 μM forskolin (forskolin) to induce syncytialisation. Membrane fusion was monitored by staining for E-cadherin (green), and cell number demonstrated with DAPI (blue). Cells were imaged at 400x magnification, scale bar: 20 μm. (**b**) Similarly cultured, transfected BeWo lines were assessed for changes in E-cadherin and syncytin expression via immunoblotting. GAPDH was used to control for protein loading and protein bands were quantified using densitometry. Data are representative of 3 independent experiments.

Another essential function for placentation is formation of the fetal-maternal interface, which is lined by multinucleate syncytiotrophoblasts resulting from membrane fusion (syncytialisation). The high expression of TPβ in the syncytium of the growth restricted placentae (Fig. 1b) strongly suggested this function would be regulated by TPβ. Treatment of BeWo cells with the TP agonist I-BOP (200 nM) did not affect basal expression of E-cadherin or syncytin by BeWo cells (Supplementary Fig. S3). Conversely, forskolin treatment (100 μM) reduced E-cadherin and augmented syncytin expression by immunofluorescence (Fig. 4a) and immunoblotting (Fig. 4b) which are both markers of syncytialisation. TPα activation on BeWo cells accelerated loss of E-cadherin (40% compared to 16% in ∅ cells; Fig. 4a,b) and enhanced syncytin expression (2.5 fold; Fig. 4b) compared to untransfected and ∅ expressing cells (Fig. 4a). TP∆^328^ did not augment E-cadherin loss but significantly increased syncytin expression (1.15 fold gain) indicating the effects of TPα on differentiation were mediated by both isoform specific (E-cadherin loss) and common (elevated syncytin expression) pathways (Fig. 4a,b). Conversely, TPβ-BeWo cells retained more E-cadherin membrane staining and higher expression by immunoblotting, with no induction of syncytin expression after forskolin treatment (Fig. 4a,b). Collectively these data suggest that TPα promotes syncytialisation during placentation while TPβ expression abrogates the process. These findings are consistent with the expression pattern of TP isoforms with high TPα expression in “normal” pregnancies promoting robust placentation while TPβ promotes insufficient placentation associated with pathological pregnancies.

### TPβ expression slows trophoblast cell migration

**Figure 5:**
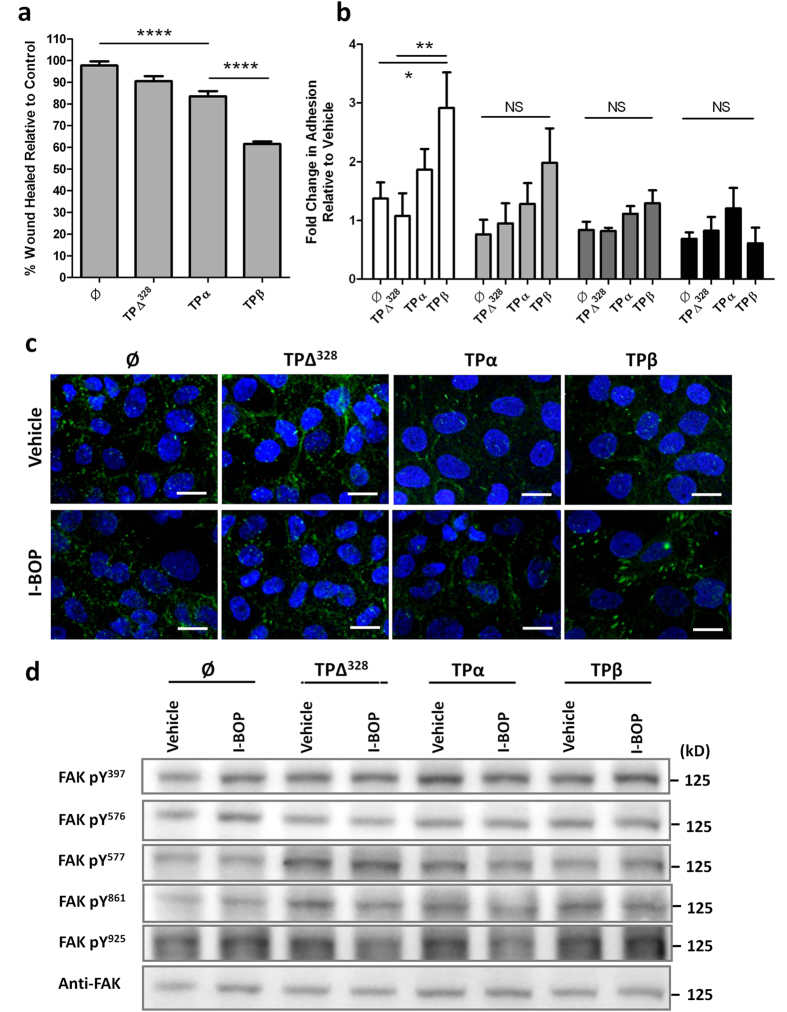
Migration of trophoblast cells is attenuated by TPβ activation. (**a**) Motility of transfected JEG-3 cells in response to I-BOP (200 nM) was investigated in a chemokinesis assay over 48 hours and compared to vehicle control. The distance the cells moved was analysed using TScratch software and expressed as a percentage wound closure, *n* = 6. (**b**) Adhesion of transfected JEG-3 cells, pre-treated with 200 nM I-BOP for 24 hours, to different extracellular matrix proteins (collagen I, □; collagen IV, 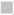; fibronectin, 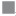; vitronectin, ■) was assessed over a 30 minute period and compared to vehicle control. Attachment was quantified using methylene blue staining. (**c**) Transfected JEG-3 cells, treated with vehicle or 200 nM I-BOP, were stained with anti-vinculin antibodies and imaged at 400x magnification via fluorescence microscopy to identify focal adhesions. Scale bar: 20 μm. (**d**) Changes in FAK activation/phosphorylation at specific residues was measured via immunoblotting in transfected JEG-3 cells treated with vehicle or 200nM I-BOP. Loading was determined by re-probing the membranes with non-phosphorylated FAK and protein bands were quantified using densitometry. The data are represented as mean ± SEM and are representative of 3 independent experiments. One-way ANOVA, NS: not significant, *P < 0.05, **P ≤ 0.01, ****P < 0.001.

A key step in the pathogenesis of asymmetric growth restriction is the invasion of the uterine wall and remodelling of spiral arteries by cytotrophoblasts. The role of TP isoforms in regulating this process is undocumented. Motility of ∅ and TPΔ^328^ transfected JEG-3 cells was unchanged, compared to untransfected cells, in the presence of I-BOP (Fig. 5a). TPα-JEG-3 cells had a mild reduction (14%) in migration compared to ∅ controls (*P* < 0.001), but not compared to TPΔ^328^-JEG-3 cells (*P* = 0.160) (Fig. 5a). TPβ overexpression reduced migration (36%) which was significant compared to ∅ (*P* < 0.001), TPΔ^328^ (*P* < 0.001) and TPα (*P* < 0.001) expressing cells (Fig. 5a).

Migration of cells is a complex process regulated by cytoskeletal dynamics, extracellular matrix (ECM) interactions and planar cell polarity. To determine the mechanism by which TPβ activation inhibited trophoblast migration we examined adhesion of cells to several matrix components. Consistent with the migration data, transfection of JEG-3 cells with ∅, TPΔ^328^ and TPα did not influence binding of trophoblasts to matrix components (Fig. 5b). Conversely, activation of TPβ enhanced JEG-3 attachment to collagen I and collagen IV based matrices (3 fold and 2.5 fold, respectively) while attachment to fibronectin and vitronectin was unchanged (Fig. 5b). This was significantly different to ∅-JEG-3 (*P* = 0.025) and TPΔ^328^-JEG-3 (*P* = 0.01) cells when binding to collagen I. Thus, the blunted migration in TPβ-JEG-3 cells might be explained by enhanced adhesion to ECM.

To further determine the role of adhesion in the inhibition of trophoblast migration by TPβ we examined focal adhesion formation in these cells. Association of vinculin with integrins denotes focal adhesion complexes actively engaged with matrix binding and is therefore implicated in the control of cell motility^36^,^37^. I-BOP treatment of TPα-JEG-3 cells (Fig. 5c) reduced the levels of vinculin staining (both size and number of focal adhesions) compared to ∅ and TPΔ^328^ transfected cells (which were no different to untransfected cells) which is indicative of fewer focal adhesions. By comparison, I-BOP treatment of TPβ-JEG-3 cells enhanced focal adhesion size 3 fold compared to TPα and TPΔ^328^ expressing cells, although focal adhesion number was maintained overall (Fig. 5c). Consistent with the migration data, there was no difference in focal adhesion size and number in TPβ cells at baseline indicating the receptor needed to be activated for these effects. The enhanced focal adhesion size in TPβ-JEG-3 cells is consistent with the greater adhesion to collagen-based matrices and suggests that enhanced adhesion to the matrix is the mechanism by which TPβ activation impedes trophoblast movement.

Focal adhesion kinase (FAK) is centrally involved in the signalling response of integrins, which interact directly with the ECM to transmit signals from the extracellular environment^38^. Consistent with the enhanced size of focal adhesions in TPβ-JEG-3 cells, TPβ activation enhanced FAK phosphorylation at Y^397^ (the FAK autophosphorylation site) up to 1.8 fold (*P* = 0.014), Y^577^ by 2 fold (*P* ≤ 0.011) and Y^861^ 1.5 fold (*P* ≤ 0.01) compared to ∅- and TPΔ^328^-JEG-3 cells (Fig. 5d). Non-significant changes in Y^576^ and Y^925^ phosphorylation were also observed in I-BOP treated TPβ-JEG-3 cells compared to the other sub-lines. Phosphorylation of Y^577^ enhances FAK activation, which would enhance focal adhesion formation, while phosphorylation of Y^861^ promotes focal adhesion targeting. The enhanced Y^577^ phosphorylation is indicative of elevated Src activation; however, activation of Src (denoted by enhanced Y^418^ and loss of Y^529^ phosphorylation) only occurred in TPα-JEG-3 cells, not TPβ-JEG-3 cells, (Supplementary Fig. S4) indicating the enhanced Y^577^ phosphorylation is likely the result of another Src family member.

### Suppression of placental and fetal growth in TPβ-transgenic mice

**Figure 6:**
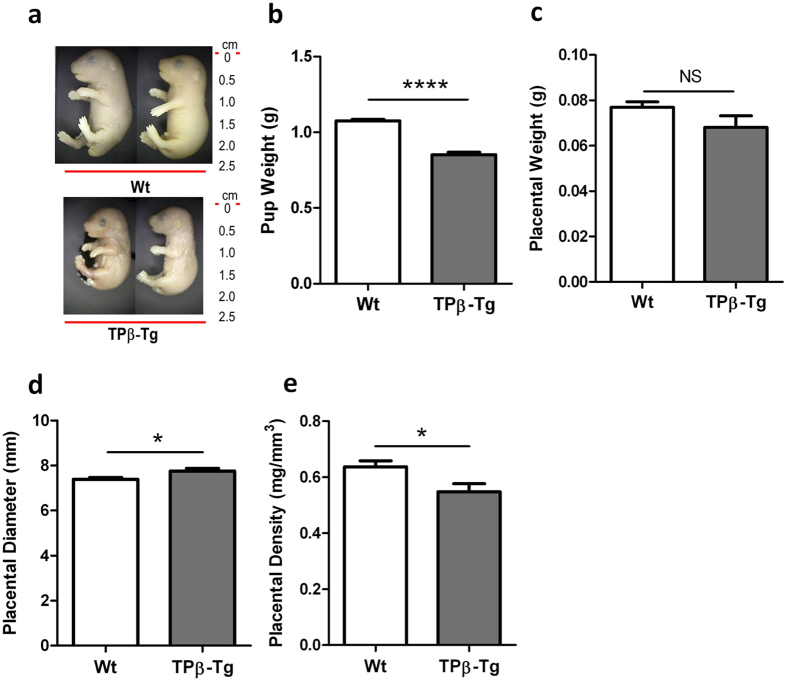
TPβ expression in mice produces a robust IUGR phenotype. (**a**) Representative images of pups born to Wt and TPβ-Tg dams at day 18.5 of gestation. All pups were born to *n* = 14 Wt and *n* = 7 TPβ-Tg dams. (**b**)Weight of pups born to Wt (□) and TPβ-Tg (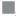) dams. Pup weight data is representative of pups (*n* = 89) born to Wt dams and pups (*n* = 48) born to TPβ-Tg dams. Weight (**c**), diameter (**d**) and density (**e**) of the placentae from *n* = 54 pups born to Wt (□) and *n* = 20 pups born to TPβ-Tg (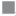) dams. The data are represented as mean ± SEM. Independent samples T-test, NS: not significant, *P < 0.05, ****P < 0.001.

Our *in vitro* data suggest that TPβ activation inhibits normal trophoblast function; however, whether expression of TPβ is a cause or a consequence of the development of IUGR remained unanswered. We characterised the TPβ-transgenic (TPβ-Tg) mice to determine a causal role for TPβ in the development of IUGR. The pups born to TPβ-Tg dams were on average 26% smaller than pups born to Wt dams (*P* < 0.001; Fig. 6a,b). In terms of assessed growth potential, 37% of all pups born to TPβ-Tg dams fell below the 10^th^ percentile rank consistent with the human classification of IUGR. There was a 12% reduction in placental weight for pups born to TPβ-Tg dams compared with the pups born to Wt dams (Fig. 6c), although this did not reach significance. Placental diameter was significantly larger in growth restricted pups born to TPβ-Tg dams (*P* = 0.014; Fig. 6d) resulting in the placentae being less dense (*P* = 0.029; Fig. 6e) as well as small. These data indicate that pups born to transgenic mice are growth restricted and not just small for gestational age.

**Figure 7:**
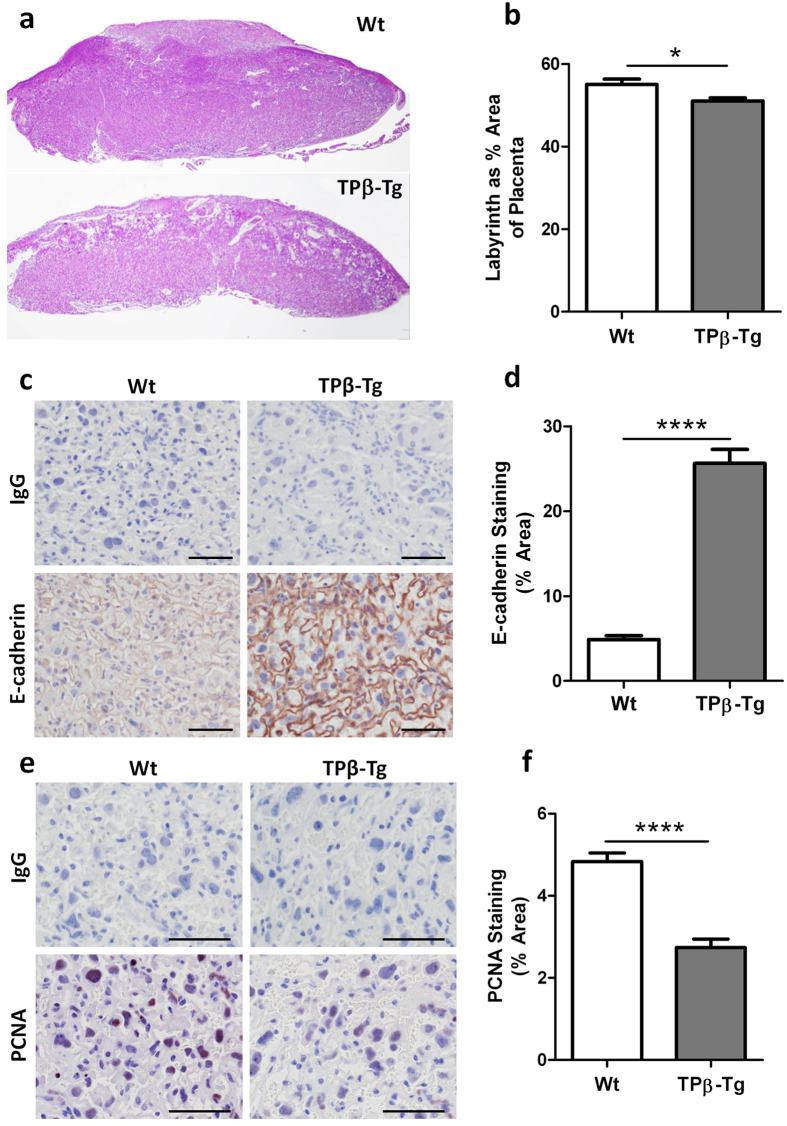
TPβ expression in mice reduces labyrinthine formation and prevents trophoblast proliferation and differentiation as part of the IUGR phenotype. (**a**) Representative images of H&E stained sections of placentae from pups born to Wt (*n* = 89) and TPβ-Tg (*n* = 48) dams at day 18.5 of gestation. (**b**) From the H&E stained sections, the size of the labyrinthine zone of placentae from pups (*n* = 9) born to Wt (□) dams and pups (*n* = 31) born to TPβ-Tg (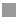) dams was elucidated using ImageJ software. Representative images (**c**) and quantification (**d**) of E-cadherin expression in the labyrinthine zone in placentae from pups (*n* = 47) born to Wt (□) dams and pups (*n* = 59) born to TPβ-Tg (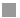) dams. Representative images (**e**) and quantification (**f**) of proliferation (PCNA staining) in the labyrinthine zone in placentae from pups (*n* = 53) born to Wt (□) dams and pups (*n* = 59) born to TPβ-Tg (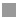) dams. All sections stained for E-cadherin and PCNA were imaged at 200x magnification, scale bar: 100 μm. The data are represented as mean ± SEM. Independent samples T-test, *P < 0.05, ****P < 0.001.

The placental pathology of the growth restricted pups born to TPβ-Tg dams was immediately obvious. Assessment of the labyrinth in the placentae from TPβ-Tg dams was 10% smaller than its Wt counterparts (*P* = 0.013; Fig. 7a,b). To explain the altered labyrinth size we documented the differentiation/syncytialisation and proliferation of trophoblasts in placentae using immunohistochemistry. Consistent with human data, Wt mouse placentae were devoid of E-cadherin staining denoting robust fusion in the syncytiotrophoblast layer (Fig. 7c,d). The area of E-cadherin expression in the labyrinth of growth restricted pups born to TPβ-Tg dams was 5.3 fold higher (Fig. 7c,d) compared to Wt mice. Moreover, the amount of proliferation, as denoted by PCNA staining, was 1.8 fold lower in the labyrinth of placentae from pups born to TPβ-Tg dams versus Wt dams (Fig. 7e,f). Together these changes in cellular function indicate that placentae of growth restricted litters from TPβ-Tg dams have reduced rates of syncytialisation and proliferation. This *in vivo* data is consistent with the abnormal function of TPβ overexpressing trophoblasts *in vitro* and clearly implicates enhanced TPβ-expression on the syncytiotrophoblasts of human placentae as causal to the development of IUGR.

## Discussion

We report that TP ligands manipulate placentation through direct actions on trophoblasts, in an isoform specific fashion. Stimulation of TPα enhanced trophoblast differentiation and proliferation while TPβ activation prevented these activities and inhibited migration. These data have important potential implications for placentation as progeny of TPβ-Tg mice displayed fetal growth restriction, accompanied by poor trophoblast proliferation and syncytialisation, and reduced placental weight. Most importantly, TPβ expression was enhanced in placental biopsies from growth restricted human pregnancies suggesting isoform switching in the pathogenesis of fetal growth restriction. The cause of idiopathic IUGR, especially asymmetrical IUGR, is unknown. The discovery of the role of the different TP isoforms in placentation, particularly that dysregulation of TPβ is observed in the growth restricted placenta, raises the possibility that this is a significant, yet targetable, pathogenic factor in human pregnancy. The acknowledgement of the role of the different TP isoforms in placentation enriches our perception of the role for TP activation in pregnancy and expands the repertoire of pathophysiological functions ascribed to the receptors.

Our observations add to the dichotomy in the literature over the role of TP isoforms in the regulation of health and disease. TPα is known to be pro-angiogenic, whilst TPβ inhibits angiogenic responses^39^. Despite these divergent findings both TP isoforms are pro-tumorigenic in epithelial cells in a tissue/organ specific fashion^40^,^41^. A previous study documented TP isoform transcripts in primary trophoblasts; however, no studies have documented TP isoform protein expression. This study showed TPα mRNA was ubiquitous and at high levels in primary trophoblasts *in vitro* but TPβ expression was more variable. However, the outcomes of these pregnancies were not known due to the trophoblasts being isolated from first trimester tissue^42^. Therefore it is possible that the trophoblasts with high TPβ expression may have been from placentae with some sort of pathology, such as IUGR. Our protein data confirms the robust expression of TPα in trophoblast cells *in vitro* and *in vivo* but we do not see the same expression of TPβ in placentae from “normal” pregnancies. TPβ expression in placentae from pathological pregnancies has not been previously documented and it is unknown how the enhanced TPβ expression results in IUGR. The control over TP splicing is likely driven by differential promoter utilization^43^; however, the lower expression of TPβ mRNA in IUGR placentae suggests that the mechanism is due to post-transcriptional regulation and protein stabilisation. Expression of TPβ, but not TPα, is increased in conditions with elevated free radical production^44^ due to both increased trafficking to the membrane and greater protein stabilization. Such changes are well established in the IUGR placenta and may be the reason for TPβ upregulation in disease.

Previous studies on the pathogenic role of TXA_2_ in IUGR were performed in small animal models^25^,^26^,^27^ where TPβ expression is absent. These data suggest that appropriate activation of TPα may promote healthy placentation and angiogenesis; however, overstimulation of TPα may result in ligand-induced receptor desensitization^45^,^46^,^47^ essentially creating an environment devoid of TP activation. The *bona fide* IUGR placenta has been reported to produce elevated TXA_2_ levels^22^,^24^ although TXA_2_ synthase levels have not been documented. Little is known about the relative importance of TP isoforms to placentation in humans or the consequences of TP activation on trophoblasts. Stimulation of primary human villous cytotrophoblasts (from term placentae) with a TXA_2_ mimetic abrogated differentiation and enhanced apoptosis and p53 expression^48^. Our data support many of these findings, including the failure to differentiate; however, we did not observe any increase in apoptosis. This may reflect differences between the low concentrations of TP agonist used here (100 nM or lower), which are more reflective of reported (patho)physiological levels^49^, versus the supra-pathological (10 μM) doses in the original study^48^. What wasn’t clear from the previous data was the TP isoform responsible for these effects. Our data clearly show that TPβ mediates the adverse effects of TXA_2_ on placentation, with neither TP∆^328^ nor TPα producing the same responses. These data further accentuate the divergent roles for TPα and TPβ as human-specific disease modifiers and emphasize the need for greater understanding of the biology of the individual isoforms. The signalling that perturbs trophoblast function and placentation resides in the divergent C-terminal residues of TPβ. Indeed, we have previously shown that the C-terminus of TPβ is all that is required for the inhibition of angiogenesis^39^. The 79 residue cytoplasmic tail of TPβ is a poor discriminator of G-protein coupling (TPα and TPβ share >70% of Gα units as second messengers) but has significantly different protein-protein interactions than that of TPα. Recently, protein-protein interactions of the tail of TPβ have been shown to manipulate the behavior of smooth muscle and cancer cells^50^,^51^ independent of G-protein coupling. However, both interactions enhance proliferation and migration in the respective cell types and interactions are yet to be identified that inhibit cellular function as described here. We believe that pathways unrelated to G-protein activation, mediated through direct interaction of proteins with the cytoplasmic tail of TPβ, regulate the negative effects of TPβ on placentation and trophoblast migration.

The primary deficit in the original report of the TPβ-Tg mice was a decrease in placental and pup size^52^; however, reductions in pup and placental weights were not quantified nor was any basis for the growth restriction explored. In the previous study only homozygous transgenic mice showed the IUGR phenotype, which could have resulted from the mixed genetic background (SV129xC57Bl/6). Our cohort have been backcrossed to C57Bl/6 for a total of 16 generations and still maintain the growth restricted phenotype suggesting that TPβ expression is the cause of the growth restriction. The trophoblast layer of these placentae was poorly formed with smaller labyrinthine zones, most likely a result of the diminished trophoblast proliferation caused by TPβ activation. This is likely to have been compounded by the anti-angiogenic effects of TPβ^39^, which would have further limited placental growth. The failure to syncytialise would have compromised fetal growth, not only by reducing the transport of nutrients and waste products of metabolism, but because the integration of fetomaternal signalling would be compromised in these placentae^53^. Again, the anti-angiogenic effects of TPβ may also play a role in fetal growth with vascular bed development possibly retarded in the TPβ-Tg pups. Thus, this model represents a unified theory for the origins of idiopathic IUGR in humans and a new target for therapeutic development.

The identification that TXA_2_ levels are elevated in IUGR, compared to “normal” pregnancies, has resulted in targeting TXA_2_ generation to provide therapeutic benefit. As in cardiovascular disease, these attempts have primarily focussed on low-dose aspirin (60–150 mg/day) to prevent TXA_2_ synthesis by platelets. Aspirin crosses the placenta and has a favourable safety profile in pregnancy^48^,^54^. Despite this, the outcomes of large scale clinical trials for the use of aspirin to prevent IUGR have been largely negative. Historically, initiation of aspirin therapy after 16 weeks gestation^53^,^54^,^55^ has been shown to be less beneficial than early in pregnancy^23^,^54^,^55^. However, whether IUGR is associated with a normotensive or hypertensive pregnancy also changes whether fetal, or maternal and fetal circulations are involved, which changes the response to aspirin therapy^41^. Moreover, aspirin appears to preferentially inhibit TXA_2_ production in villous cell types other than the trophoblast^45^. Our data would suggest that all these factors are issues in the effectiveness of aspirin therapy as TPβ is activated not just in response to TXA_2_ but also isoprostanes^18^. Administration of aspirin to promote appropriate placentation, and negate the effects of TPβ, would only be effective early in pregnancy and may need to target both maternal and fetal circulations, as TPβ is expressed on both fetal and maternal endothelial cells and syncytiotrophoblasts. As such, current therapies for IUGR are insufficient as they do not appropriately target TPβ activation, resulting in persistent disease. Conversely, the use of dual action TP/TXA_2_ synthase antagonists has not been trialled in IUGR, although these drugs are effective in animal models of IUGR^31^, and may represent a better therapeutic option than aspirin.

Collectively our data suggest that the upregulation of TPβ in placentae of IUGR pregnancies initiates a cascade which compromises trophoblast proliferation, invasive potential and differentiation, to produce an environment unable to support placental growth and fetal development. Our data are the first to elucidate the role of the human-specific isoform of TP, TPβ, as a pathogenic component in the development of IUGR and suggests that targeting TPβ activation, or TP splicing, offers a therapeutic avenue for IUGR, which currently has no effective therapies.

## Materials and Methods

### Materials

The TXA_2_ mimetic I-BOP ([1S-[1α,2α(Z),3β(1E,3S*),4α]]-7-[3-[3-hydroxy-4-(4-iodophenoxy)-1-butenyl]-7-oxabicyclo[2.2.1]hept-2-yl]-5-heptenoic acid) was from Cayman Chemical. Forskolin and L-Mimosine were from Sigma Aldrich. All cell culture reagents were from Life Technologies, immunohistochemical regents from DAKO and general chemicals from Sigma-Aldrich unless otherwise stated.

### Tissue collection

This study was performed in accordance with the guidelines approved by the Northern Sydney Local Health District Human Research Ethics Committee, St Leonards, NSW, Australia and was assigned the site specific assessment number 0912–348M and the Australian national ethics application form number HREC/09/HARBR/165. Collection of human placental tissue was performed with written informed consent obtained prior to delivery. Placentae were collected from non-labouring or minimally labouring women at delivery by lower caesarean section from pregnancies complicated by IUGR or gestationally matched control pregnancies. Patient populations were matched for body mass index (BMI) and smoking, gestational diabetes, pre-existing hypertension and pre-eclampsia were exclusion criteria (Table 1). IUGR was diagnosed prior to delivery by ultrasound, where estimated fetal weight of less than the 10^th^ percentile for gestational age was associated with elevated fetoplacental vascular resistance defined as the Systolic/Diastolic ratio over the 95^th^ percentile or absent or reversed flow in diastole in the umbilical artery, and confirmed by weight at birth. Placental tissue was washed in sterile saline and either snap frozen in liquid nitrogen or fixed in 10% (v/v) neutral buffered formalin prior to paraffin embedding or extraction of protein/RNA.

### Cell culture and transfection with vectors encoding TP isoforms

Choriocarcinoma cell lines (BeWo and JEG-3) were maintained in RPMI culture medium supplemented with 10% (v/v) fetal calf serum, 2 mM L-glutamine, 10 μg/mL penicillin and 10 U/mL streptomycin. Sub-confluent monolayers were passaged weekly using TrypLE Express. To ensure maximal TP activation, BeWo and JEG-3 cells were treated with 200 nM I-BOP. Syncytialisation of BeWo cells was induced by treating with 100 μM forskolin^56^.

The coding sequence for TPα, TPβ, and the deletion mutant (TPΔ^328^) were cloned into pcDNA3.1. TP receptor constructs were introduced into BeWo and JEG-3 cells using Effectene (Qiagen Pty Ltd.) or Lipofectamine 2000 (Life Technologies) transfection reagents, respectively, following the manufacturers’ instructions. Empty vector (∅) was used as a control. Stable expression was achieved in BeWo and JEG-3 cells after antibiotic selection using 200 μg/mL and 400 μg/mL Geneticin (G418 sulfate), respectively. Receptor expression was confirmed using semi-quantitative RT-PCR on RNA from stably expressing cells.

### Quantitative gene expression using Droplet Digital PCR

RNA isolation was performed using TRIzol Reagent according to the manufacturer’s recommendations. Tissue samples were weighed, placed into an M-tube with the appropriate amount of TRIzol Reagent and disaggregated using a gentleMACS Dissociator (Miltenyi Biotec). Cell lines were scraped directly into TRIzol Reagent. RNA quality and concentration were assessed using a NanoDrop 1000 Spectrophotometer (Thermo Fisher Scientific) and subsequently 1 μg RNA was reverse transcribed to cDNA using BioScript Reverse Transcriptase (Bioline). Gene expression was quantitated using the QX200 Droplet Digital PCR (ddPCR) System (Bio-Rad). Reactions were prepared using the QX200 ddPCR EvaGreen Supermix, 50 ng cDNA and the following primers which span the intron-exon boundary responsible for alternative splicing of TP or an internal control gene (HPRT1):

TP (Total) Forward 5′-CGGGTTCAAGCGATTCTCG-3′

TP (Total) Reverse 5′-CCTGTTGGAGGTTCAAAAGGAAG-3′

TPα Forward 5′-CGCACCACGGAGAAGGAG-3′

TPα Reverse 5′-CTGGGGCTGGAGGGACAG-3′

TPβ Forward 5′-CTTCTGGTCTTCATCGCC-3′

TPβ Reverse 5′-AAAGGAAGCAACTGTACCCC-3′

HPRT1 Forward (internal control) 5′-GTTTGTTGTAGGATATGCCCTTGAC-3′

HPRT1 Reverse 5′-GACTCCAGATGTTTCCAAACTCAAC-3′

Droplets were generated from the reactions using QX200 Droplet Generation Oil for EvaGreen on the QX200 Droplet Generator (Bio-Rad) according to the manufacturer’s instructions. The droplets were cycled on a T100 Gradient Thermal Cycler (Bio-Rad) according to the manufacturer’s protocol using an annealing temperature of 60 °C. The droplets were analysed using the QX200 Droplet Reader and analysed using QuantaSoft Software (Bio-Rad). Detection of <15,000 droplets in a sample indicated poor quality and was therefore excluded from the analysis.

### Chemokinesis assay

JEG-3 cells were chosen as BeWo cells did not migrate well in these assays. Transfected JEG-3 cells were grown to confluence and motility assessed as previously described^39^ in the presence of I-BOP (200 nM) or vehicle control (equivalent volume of ethanol). Media was supplemented with L-Mimosine (400 μM) to negate the effects of proliferation on the outcome of the assay. Photographs were taken at the time of injury (0 hours) and after 24 and 48 hours. T-Scratch software was used to quantify the distance migrated and the migration of I-BOP treated cells was then normalised relative to vehicle control treated cells.

### Adhesion assay

Adhesion of JEG-3 cells to matrix proteins, including collagen I (Life Technologies), collagen IV (Sigma-Aldrich), fibronectin (Life Technologies) and vitronectin (Promega) was assessed as previously described^39^. JEG-3 cells were cultured in the presence of 200 nM I-BOP, or vehicle control, for 24 hours prior to the assay. Cells were detached using TrypLE Express, plated into pre-coated 24 well plates at 1 × 10^5^ cells/well and incubated for 37 °C for 30 minutes. After washing to remove unattached cells, wells were fixed with 10% (v/v) neutral buffered formalin and adhesion detected by staining with 1% (w/v) methylene blue as previously described^57^.

### Cell proliferation, apoptosis and cell cycle analysis by flow cytometry

JEG-3 and BeWo cell proliferation was assessed by seeding 2 × 10^4^ cells/well into 24 well plates. Cells were cultured in the presence of 200 nM I-BOP, or vehicle control, for up to 7 days. Cells were enumerated by cell counting or staining with 1% (w/v) methylene blue. Cell cycle and apoptosis analyses were performed on randomly cycling, and growth arrested, BeWo cells as previously described^58^. DNA content was analysed using a BD FACSCalibur Flow Cytometer and ModFit software. Apoptosis was quantified as the hypodiploid peak in these samples.

### SDS PAGE, slot blots and immunoblotting

Protein lysates from transfected BeWo and JEG-3 cell lines were obtained as described^59^. For placental tissue, biopsies were weighed, placed into an M-tube with the appropriate amount of lysis buffer containing 1% (v/v) Y30 and disaggregated using a gentleMACS Dissociator (Miltenyi Biotec). For phospho-proteins, lysates were prepared using hot SDS-PAGE buffer as previous described^58^. Concentration of protein lysates was estimated using the Pierce BCA Protein Assay Kit (Life Technologies) and 30 μg separated by SDS-PAGE under reducing conditions on 8%-10% (w/v) polyacrylamide gels. After transfer to PVDF membranes, immunoblotting was performed as previously described^39^ for: Cyclin A, B1, D1, E, p16, p21, p27, p53 (1:1000, Santa Cruz Biotechnology) and Retinoblastoma Protein (Rb) (pSer^780^, pSer^795^ and pSer^807/811^; 1:1000, Cell Signaling Technology), E-cadherin and syncytin (Santa Cruz Biotechnology), FAK (pY^397^, pY^576/577^, pY^925^; 1:2000, Life Technologies) and Src (pY^418^, pY^529^; 1:2000, Life Technologies). Total protein content was assessed by immunoblotting for GAPDH (Santa Cruz Biotechnology) or unphosphorylated versions of the proteins of interest (FAK/Src/Rb; 1:2000, Santa Cruz Biotechnology and Cell Signaling Technology).

For slot blotting, 100 μg of protein lysate from placental samples was loaded into a manifold (Bio-Rad) and adsorbed onto nitrocellulose membranes under vacuum. Membranes were probed for total TP content (Santa Cruz Biotechnology), TPα, TPβ (using our in-house antibodies)^40^ and loading for cellular content was assessed using a Histone H1 antibody (1:1000, Santa Cruz Biotechnology). Protein expression by immunoblotting was detected using the appropriate HRP-conjugated secondary antibodies (1:3000, Dako) in combination with an enhanced chemiluminescence detection system (PerkinElmer) and ImageQuant LAS 4000 (GE Healthcare). Densitometry of protein bands were quantitated using ImageJ software.

### Immunofluorescent imaging for cell membrane structure and focal adhesion complexes

Cells were plated onto glass coverslips pre-coated with 0.2% (w/v) gelatine and 0.2 μg/mL fibronectin. For syncytialisation assays BeWo cell culture medium was supplemented with 200 nM I-BOP, with or without 100 μM forskolin, and replenished every 24 hours over 3 days. For detection of focal adhesions, JEG-3 cells were exposed to 200 nM I-BOP, or vehicle control, for 24 hours. Immunostaining after fixation in 4% (w/v) paraformaldehyde was performed as previously described^39^, which involved incubation with antibodies against E-cadherin (1:500, Santa Cruz Biotechnology) or vinculin (Sigma Aldrich) and detected using an Alexa Fluor 488 conjugated second antibody (1:800; Molecular Probes). Coverslips were mounted onto glass slides and counterstained with DAPI. Slides were imaged using an Olympus DP71 fluorescent microscope at 400x magnification using Olympus DP controller software and then images were overlayed using ImageJ software.

### Estimates of placental structure in TPβ-Tg and wild-type mice

The animal component of this study was performed in accordance with the guidelines approved by the Kolling Institute of Medical Research’s Animal Ethics Committee, St Leonards, NSW, Australia and was assigned the protocol number 1311–015A. TPβ-transgenic (TPβ-Tg) mice^52^ were bred from their original mixed C57Bl6/SV129 background to be congenic on the C57Bl/6J background (>15 generations). Mice were bred as hemizygote crosses with wild-type (Wt) C57Bl/6J mice. Mice were housed within the Kearns facility at the Kolling Institute within individually vented cages under PC2 conditions and had food and water *ad libitum*. Timed intra-strain mating of Wt and TPβ-Tg mice (10–12 weeks old) were performed using nulliporous female mice. Females with copulation plugs were separated at E0.5 and pregnancies carried until E18.5 when mice were euthanized using inhalation anaesthetic, tissues collected and biometrics on the pups assessed. The cohort of mice included 89 pups born to 14 Wt dams and 48 pups born to 7 TPβ-Tg dams. Quantification of labyrinthine area was performed using ImageJ software.

### Immunohistochemistry

Formalin fixed, paraffin embedded human and mouse placental tissues were sectioned (4 μm), dewaxed and rehydrated through graded alcohols. Haematoxylin and eosin (H&E) staining was performed to identify placental structure. Immunohistochemistry (IHC) was performed as previously described^60^ with antigen retrieval performed at 99 °C for 20 minutes in EnVision FLEX retrieval solution (high pH, Dako). Slides were incubated in sequenza racks (Thermo Fisher Scientific) overnight at 4 °C with our primary antibodies against TPα and TPβ (1:400; raised in-house)^40^, E-cadherin (2 μg/mL; Cell Signaling Technology) and PCNA (1 μg/mL; Santa Cruz Biotechnology) or anti-rabbit IgG isotype control at equivalent concentrations. NovaRed peroxidase HRP substrate kit (Vector Laboratories) was used to visualise the staining following the manufacturer’s instructions. Sections were counterstained using Mayer’s haematoxylin (Merck Millipore) and mounted with Eukitt mounting medium (Grale HDS). All placental sections were imaged using a Nikon ECLIPSE 80i light microscope with a Nikon Digital Sight Control Unit and Nikon Digital Sight DS-5M camera at 200x magnification using NIS-Elements software version 3.

### Statistical analyses

Statistical analyses based on One-way analysis of variance (ANOVA) were performed using SPSS (version 20). LSD or Games-Howell post-hoc tests were used to analyse densitometry readings from western blot analyses and percentage wound closure in scratch assays. Cell proliferation data were assessed using a general linear model analysis as well as linear contrasts within the program R. All other data was analysed using Independent samples T-tests or Mann-Whitney U non-parametric tests using SPSS. All data are displayed as mean ± standard error of the mean (SEM).

## Additional Information

**How to cite this article**: Powell, K. L. *et al*. Role for the thromboxane A_2_ receptor β-isoform in the pathogenesis of intrauterine growth restriction. *Sci. Rep*. **6**, 28811; doi: 10.1038/srep28811 (2016).

## Supplementary Material

Supplementary Information

## Figures and Tables

**Table 1:** Clinical data on placental samples from “normal” and IUGR affected pregnancies.

Subgroup	Maternal Age	BMI	SBP (mm/Hg)	DBP (mm/Hg)	Gestational Age at Delivery (weeks)	Birth Weight (g)	Fetal Gender (M/F)
Control (*n* = 53)	31.5 (5.0)*	24.9 (6.1)	113.5 (12.4)	67.6 (10.7)*	34.0 (4.4)	2443.0 (989.2)***	67.9%/32.1%
IUGR (*n* = 22)	34.1 (3.9)	22.6 (3.2)	119.6 (14.7)	75.2 (10.1)	35.6 (2.9)	1745.0 (632.3)	45.4%/54.5%

Clinical characteristics are shown for the control (gestationally matched) and IUGR patients recruited for placental sample collection. Data include maternal age, body mass index (BMI), systolic (SBP) and diastolic (DBP) blood pressure, gestational age at delivery, birth weight and fetal gender. Data are represented as mean (SD) (*n* = 53 and 22). Independent samples T-test, *P < 0.05, ***P < 0.005.

## References

[b1] Unterscheider J. . Optimizing the definition of intrauterine growth restriction: the multicenter prospective PORTO Study. Am. J. Obstet. Gynecol. 208, 290 e291–e296 (2013). 23531326 10.1016/j.ajog.2013.02.007

[b2] Bamfo J. E. & Odibo A. O. Diagnosis and management of fetal growth restriction. J. Pregnancy 2011, 640715 (2011). 21547092 10.1155/2011/640715 PMC3087156

[b3] Mahale N. . Doppler prediction of adverse perinatal outcome in intrauterine growth restriction. Int. J. Reprod. Contracept. Obstet. Gynecol. 4, 119–130 (2015).

[b4] Bernstein I. M., Horbar J. D., Badger G. J., Ohlsson A. & Golan A. Morbidity and mortality among very-low-birth-weight neonates with intrauterine growth restriction. Am. J. Obstet. Gynecol. 182, 198–206 (2000). 10649179 10.1016/s0002-9378(00)70513-8

[b5] Garite T. J., Clark R. & Thorp J. A. Intrauterine growth restriction increases morbidity and mortality among premature neonates. Am. J. Obstet. Gynecol. 191, 481–487 (2004). 15343225 10.1016/j.ajog.2004.01.036

[b6] Pallotto E. K. & Kilbride H. W. Perinatal outcome and later implications of intrauterine growth restriction. Clin. Obstet. Gynecol. 49, 257–269 (2006). 16721105 10.1097/00003081-200606000-00008

[b7] Chan P. Y., Morris J. M., Leslie G. I., Kelly P. J. & Gallery E. D. The long-term effects of prematurity and intrauterine growth restriction on cardiovascular, renal, and metabolic function. Int. J. Pediatr. 2010, 280402 (2010). 21197428 10.1155/2010/280402 PMC3010629

[b8] Conde-Agudelo A., Papageorghiou A. T., Kennedy S. H. & Villar J. Novel biomarkers for predicting intrauterine growth restriction: a systematic review and meta-analysis. BJOG. 120, 681–694 (2013). 23398929 10.1111/1471-0528.12172

[b9] Nardozza L. M. . Fetal growth restriction: current knowledge to the general Obs/Gyn. Arch. Gynecol. Obstet. 286, 1–13 (2012). 22526452 10.1007/s00404-012-2330-6

[b10] Macara L. . Structural analysis of placental terminal villi from growth-restricted pregnancies with abnormal umbilical artery Doppler waveforms. Placenta. 17, 37–48 (1996). 8710812 10.1016/s0143-4004(05)80642-3

[b11] Chen C. P., Bajoria R. & Aplin J. D. Decreased vascularization and cell proliferation in placentas of intrauterine growth-restricted fetuses with abnormal umbilical artery flow velocity waveforms. Am. J. Obstet. Gynecol. 187, 764–769 (2002). 12237661 10.1067/mob.2002.125243

[b12] Mayhew T. M. . Stereological investigation of placental morphology in pregnancies complicated by pre-eclampsia with and without intrauterine growth restriction. Placenta. 24, 219–226 (2003). 12566249 10.1053/plac.2002.0900

[b13] Sato Y. . Associations of intrauterine growth restriction with placental pathological factors, maternal factors and fetal factors; clinicopathological findings of 257 Japanese cases. Histol. Histopathol. 28, 127–132 (2013). 23233065 10.14670/HH-28.127

[b14] Chaddha V., Viero S., Huppertz B. & Kingdom J. Developmental biology of the placenta and the origins of placental insufficiency. Semin. Fetal Neonatal Med. 9, 357–369 (2004). 15691771 10.1016/j.siny.2004.03.006

[b15] Boulot P., Giacalone P. L., Hedon B., Laffargue F. & Viala J. L. Fetal growth retardation: physiopathology. Review of the literature. J. Gynecol. Obstet. Biol. Reprod. (Paris) 21, 851–856 (1992). 1491129

[b16] Kaufmann P., Black S. & Huppertz B. Endovascular trophoblast invasion: implications for the pathogenesis of intrauterine growth retardation and preeclampsia. Biol. Reprod. 69, 1–7 (2003). 12620937 10.1095/biolreprod.102.014977

[b17] Roberts L. J. & Morrow J. D. Measurement of F(2)-isoprostanes as an index of oxidative stress *in vivo*. Free Radic. Biol. Med. 28, 505–513 (2000). 10719231 10.1016/s0891-5849(99)00264-6

[b18] Audoly L. P. . Cardiovascular responses to the isoprostanes iPF(2alpha)-III and iPE(2)-III are mediated via the thromboxane A(2) receptor *in vivo*. Circulation. 101, 2833–2840 (2000). 10859290 10.1161/01.cir.101.24.2833

[b19] Montuschi P., Barnes P. J. & Roberts L. J. 2nd. Isoprostanes: markers and mediators of oxidative stress. FASEB J. 18, 1791–1800 (2004). 15576482 10.1096/fj.04-2330rev

[b20] Sorem K. A. & Siler-Khodr T. M. Effect of IGF-I on placental thromboxane and prostacyclin release in severe intrauterine growth retardation. J. Matern. Fetal. Med. 6, 341–350 (1997). 9438218 10.1002/(SICI)1520-6661(199711/12)6:6<341::AID-MFM9>3.0.CO;2-O

[b21] Xu J., Ma T. & Wen L. Study on relativity between oxygen free radical and thromboxane B2, 6-keto-PGF1 alpha during ligustrazine treatment of intrauterine growth retardation. Zhongguo Zhong Xi Yi Jie He Za Zhi. 18, 265–268 (1998). 11477921

[b22] Lynch C. M. . The role of thromboxane A(2) in the pathogenesis of intrauterine growth restriction associated with maternal smoking in pregnancy. Prostaglandins Other Lipid Mediat. 95, 63–67 (2011). 21723954 10.1016/j.prostaglandins.2011.06.007

[b23] Davi G. . Thromboxane biosynthesis and platelet function in type II diabetes mellitus. N. Engl. J. Med. 322, 1769–1774 (1990). 2345567 10.1056/NEJM199006213222503

[b24] Siler-Khodr T. M. . Effect of ethanol on thromboxane and prostacyclin production in the human placenta. Alcohol. 21, 169–180 (2000). 10963940 10.1016/s0741-8329(00)00084-7

[b25] Fung C. . Novel thromboxane A2 analog-induced IUGR mouse model. J. Dev. Orig. Health Dis. 2, 291–301 (2011). 25141265 10.1017/S2040174411000535

[b26] Hayakawa M., Mimura S., Sasaki J. & Watanabe K. Neuropathological changes in the cerebrum of IUGR rat induced by synthetic thromboxane A2. Early Hum. Dev. 55, 125–136 (1999). 10390088 10.1016/s0378-3782(99)00023-7

[b27] Hayakawa M. . Carbohydrate and energy metabolism in the brain of rats with thromboxane A2-induced fetal growth restriction. Pediatr. Res. 70, 21–24 (2011). 21436760 10.1203/PDR.0b013e31821b9d7c

[b28] Hayakawa M. . An animal model of intrauterine growth retardation induced by synthetic thromboxane a(2). J. Soc. Gynecol. Investig. 13, 566–572 (2006). 10.1016/j.jsgi.2006.09.007 17110136

[b29] Sasaki J. . Abnormal cerebral neuronal migration in a rat model of intrauterine growth retardation induced by synthetic thromboxane A(2). Early Hum. Dev. 58, 91–99 (2000). 10854796 10.1016/s0378-3782(00)00069-4

[b30] Verlohren S. . Uterine vascular function in a transgenic preeclampsia rat model. Hypertension. 51, 547–553 (2008). 18195162 10.1161/HYPERTENSIONAHA.107.103176

[b31] Tanaka M. . Effects of a thromboxane synthetase inhibitor (OKY-046) in an ischemia-reperfusion model of intrauterine growth retardation in Sprague-Dawley rats. Biol. Neonate. 72, 181–186 (1997). 9303217 10.1159/000244482

[b32] Hirata M. . Cloning and expression of cDNA for a human thromboxane A2 receptor. Nature. 349, 617–620 (1991). 1825698 10.1038/349617a0

[b33] Raychowdhury M. K. . Alternative splicing produces a divergent cytoplasmic tail in the human endothelial thromboxane A2 receptor. J. Biol. Chem. 269, 19256–19261 (1994). 8034687

[b34] Hirata T., Ushikubi F., Kakizuka A., Okuma M. & Narumiya S. Two thromboxane A2 receptor isoforms in human platelets. Opposite coupling to adenylyl cyclase with different sensitivity to Arg60 to Leu mutation. J. Clin. Invest. 97, 949–956 (1996). 8613548 10.1172/JCI118518 PMC507140

[b35] Kinsella B. T. Thromboxane A2 signalling in humans: a ‘Tail’ of two receptors. Biochem. Soc. Trans. 29, 641–654 (2001). 11709048 10.1042/0300-5127:0290641

[b36] Cohen D. M., Kutscher B., Chen H., Murphy D. B. & Craig S. W. A conformational switch in vinculin drives formation and dynamics of a talin-vinculin complex at focal adhesions. J. Biol. Chem. 281, 16006–16015 (2006). 16608855 10.1074/jbc.M600738200

[b37] Humphries J. D. . Vinculin controls focal adhesion formation by direct interactions with talin and actin. J. Cell Biol. 179, 1043–1057 (2007). 18056416 10.1083/jcb.200703036 PMC2099183

[b38] Cooper L. A., Shen T. L. & Guan J. L. Regulation of focal adhesion kinase by its amino-terminal domain through an autoinhibitory interaction. Mol. Cell. Biol. 23, 8030–8041 (2003). 14585964 10.1128/MCB.23.22.8030-8041.2003 PMC262338

[b39] Ashton A. W. & Ware J. A. Thromboxane A2 receptor signaling inhibits vascular endothelial growth factor-induced endothelial cell differentiation and migration. Circ. Res. 95, 372–379 (2004). 15242977 10.1161/01.RES.0000138300.41642.15

[b40] Moussa O. . Novel role of thromboxane receptors beta isoform in bladder cancer pathogenesis. Cancer Res. 68, 4097–4104 (2008). 18519668 10.1158/0008-5472.CAN-07-6560

[b41] Wei J., Yan W., Li X., Ding Y. & Tai H. H. Thromboxane receptor alpha mediates tumor growth and angiogenesis via induction of vascular endothelial growth factor expression in human lung cancer cells. Lung Cancer 69, 26–32 (2010). 19853959 10.1016/j.lungcan.2009.09.009

[b42] Miggin S. M. & Kinsella B. T. Expression and tissue distribution of the mRNAs encoding the human thromboxane A2 receptor (TP) alpha and beta isoforms. Biochim. Biophys. Acta. 1425, 543–559 (1998). 9838218 10.1016/s0304-4165(98)00109-3

[b43] Coyle A. T. & Kinsella B. T. Characterization of promoter 3 of the human thromboxane A receptor gene. A functional AP-1 and octamer motif are required for basal promoter activity. FEBS J. 272, 1036–1053 (2005). 15691336 10.1111/j.1742-4658.2004.04538.x

[b44] Valentin F., Field M. C. & Tippins J. R. The mechanism of oxidative stress stabilization of the thromboxane receptor in COS-7 cells. J. Biol. Chem. 279, 8316–8324 (2004). 14583632 10.1074/jbc.M306761200

[b45] Yan F. X., Yamamoto S., Zhou H. P., Tai H. H. & Liao D. F. Serine 331 is major site of phosphorylation and desensitization induced by protein kinase C in thromboxane receptor alpha. Acta Pharmacol. Sin. 23, 952–960 (2002). 12370102

[b46] Spurney R. F. Regulation of thromboxane receptor (TP) phosphorylation by protein phosphatase 1 (PP1) and PP2A. J. Pharmacol. Exp. Ther. 296, 592–599 (2001). 11160648

[b47] Yamamoto S., Yan F., Zhou H. & Tai H. H. Agents that elevate cyclic AMP induce receptor phosphorylation primarily at serine 331 in HEK 293 cells overexpressing human thromboxane receptor alpha. Biochem. Pharmacol. 64, 375–383 (2002). 12147288 10.1016/s0006-2952(02)01053-5

[b48] Yusuf K. . Thromboxane A(2) limits differentiation and enhances apoptosis of cultured human trophoblasts. Pediatr. Res. 50, 203–209 (2001). 11477204 10.1203/00006450-200108000-00007

[b49] Karner G. & Perktold K. The Influence of Flow on the Concentration of Platelet Active Substances in the Vicinity of Mural Microthrombi. Comput. Methods Biomech. Biomed. Engin. 1, 285–301 (1998). 11264810 10.1080/01495739808936708

[b50] Louden K. A. . Neonatal platelet reactivity and serum thromboxane B2 production in whole blood: the effect of maternal low dose aspirin. Br. J. Obstet. Gynaecol. 101, 203–208 (1994). 8193093 10.1111/j.1471-0528.1994.tb13110.x

[b51] Turner E. C. . Identification of an interaction between the TPalpha and TPbeta isoforms of the human thromboxane A2 receptor with protein kinase C-related kinase (PRK) 1: implications for prostate cancer. J. Biol. Chem. 286, 15440–15457 (2011). 21357687 10.1074/jbc.M110.181180 PMC3083147

[b52] Rocca B. . Directed vascular expression of the thromboxane A2 receptor results in intrauterine growth retardation. Nat. Med. 6, 219–221 (2000). 10655114 10.1038/72334

[b53] Newnham J. P., Godfrey M., Walters B. J., Phillips J. & Evans S. F. Low dose aspirin for the treatment of fetal growth restriction: a randomized controlled trial. Aust. N. Z. J. Obstet. Gynaecol. 35, 370–374 (1995). 8717556 10.1111/j.1479-828x.1995.tb02144.x

[b54] Leitich H., Egarter C., Husslein P., Kaider A. & Schemper M. A meta-analysis of low dose aspirin for the prevention of intrauterine growth retardation. Br. J. Obstet. Gynaecol. 104, 450–459 (1997). 9141582 10.1111/j.1471-0528.1997.tb11497.x

[b55] Bujold E. . Prevention of preeclampsia and intrauterine growth restriction with aspirin started in early pregnancy: a meta-analysis. Obstet. Gynecol. 116, 402–414 (2010). 20664402 10.1097/AOG.0b013e3181e9322a

[b56] Pathirage N. A. . Homeobox gene transforming growth factor beta-induced factor-1 (TGIF-1) is a regulator of villous trophoblast differentiation and its expression is increased in human idiopathic fetal growth restriction. Mol. Hum. Reprod. 19, 665–675 (2013). 23761267 10.1093/molehr/gat042

[b57] Oliver M. H., Harrison N. K., Bishop J. E., Cole P. J. & Laurent G. J. A rapid and convenient assay for counting cells cultured in microwell plates: application for assessment of growth factors. J. Cell Sci. 92 (Pt 3), 513–518 (1989). 2592453 10.1242/jcs.92.3.513

[b58] Ashton A. W. . Inhibition of endothelial cell migration, intercellular communication, and vascular tube formation by thromboxane A(2). J. Biol. Chem. 274, 35562–35570 (1999). 10585431 10.1074/jbc.274.50.35562

[b59] Ashton A. W., Ware G. M., Kaul D. K. & Ware J. A. Inhibition of tumor necrosis factor alpha-mediated NFkappaB activation and leukocyte adhesion, with enhanced endothelial apoptosis, by G protein-linked receptor (TP) ligands. J. Biol. Chem. 278, 11858–11866 (2003). 12517920 10.1074/jbc.M210766200

[b60] Woolnough C. . Source of angiopoietin-2 in the sera of women during pregnancy. Microvasc. Res. 84, 367–374 (2012). 22995870 10.1016/j.mvr.2012.08.003

