# Supplementary material for: Role for the thromboxane A2 receptor β-isoform in the pathogenesis of intrauterine growth restriction
Source: Sci Rep. 2016 Jul 1;6:28811. doi: 10.1038/srep28811 (PMC4929481; doi:10.1038/srep28811)
Supplement: Supplementary Information [file srep28811-s1.pdf]

Role for the thromboxane A_2 receptor β-isoform in the pathogenesis of intrauterine growth restriction

Authors: Katie L Powell[1,2,3]*, Veronica Stevens[1,2]*, Dannielle H Upton[1,4], Sharon A McCracken[1,2], Ann M Simpson[4,5], Yan Cheng[6], Vitomir Tasevski[1,3,4], Jonathan M Morris[1,2], Anthony W Ashton[1,2]

Affiliation:

[1]Division of Perinatal Research, Kolling Institute, Northern Sydney Local Health District, St Leonards, NSW, 2065, Australia.

[2]Sydney Medical School Northern, University of Sydney, NSW, 2006, Australia.

[3]Pathology North, NSW Health Pathology, Royal North Shore Hospital, St Leonards, NSW, 2065, Australia.

[4]School of Life Sciences, University of Technology Sydney, Ultimo, NSW, 2007, Australia.

[5]Centre for Health Technologies, University of Technology Sydney, Ultimo, NSW, 2007, Australia.

[6]Institute for Translational Medicine and Therapeutics, University of Pennsylvania School of Medicine, Philadelphia, Pennsylvania, 19104, USA.

* These authors contributed equally to the work

Supplementary Figure 1

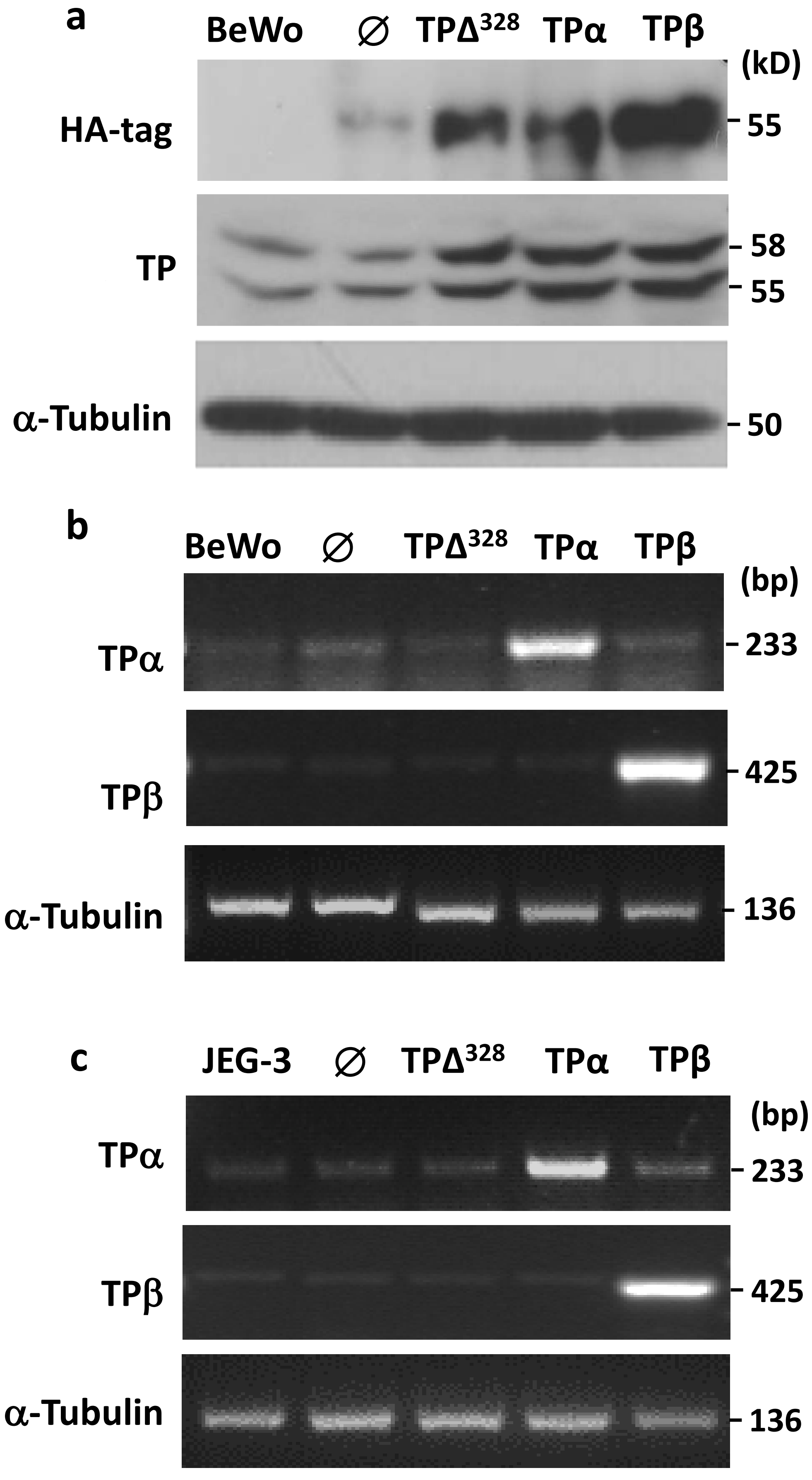

Supplementary Figure 1. Expression of TP isoforms in transfected BeWo and JEG-3 cell lines. (**a**) TP isoform expression was confirmed by immunoblotting for both the N-terminal HA-Tag and common domain of the receptor and expression was compared to α-Tubulin (loading control), *n*=6. Expression of the individual TP isoforms in transfected BeWo (**b**) and JEG-3 (**c**) cell lines was confirmed using RT-PCR and α-Tubulin was used as a positive control. *n*=5 and n=6, respectively.

Supplementary Figure 2

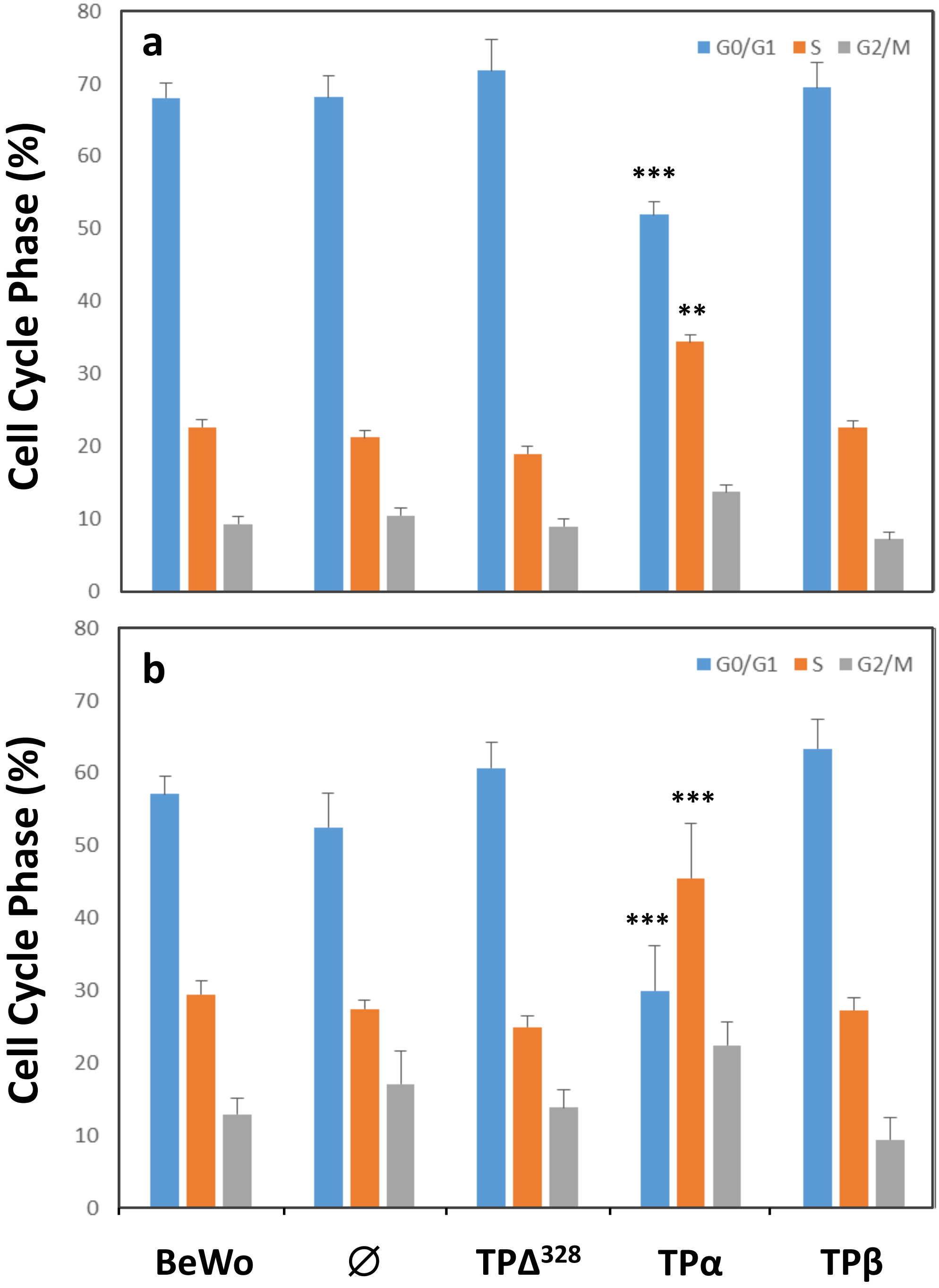

Supplementary Figure 2: Cell cycle kinetics of trophoblasts are differentially regulated by TP isoforms. (**a**) Untransfected BeWo cells and those transfected with empty vector (∅), $TP\Delta^{328}$, $TP\alpha$ and $TP\beta$ were synchronised with 400 mM L-Mimosine and the ability to void cell cycle arrest (determined by percentage of cells in G_0/G_1 phase) was assessed by flow cytometry. BeWo-$TP\alpha$ cells do not arrest to the same degree as other BeWo lines with higher S-phase and lower G_0/G_1 phase populations. (**b**) Re-entry of arrested cells into the cell cycle (as assessed by proportion of cells in S-phase) was assessed by flow cytometry 24 hours after removal of L-Mimosine. BeWo-$TP\alpha$ cells re-enter the cell cycle more quickly than other BeWo lines with higher S-phase and G_2/M, with lower G_0/G_1, phase populations. Conversely, BeWo-$TP\beta$ re-enter the cell cycle more slowly than BeWo-∅ or BeWo-$TP\Delta^{328}$ cells. The data are represented as mean ± SEM for n=6 experiments. Independent samples T-test, ** $P<0.01$, *** $P<0.005$.

Supplementary Figure 3

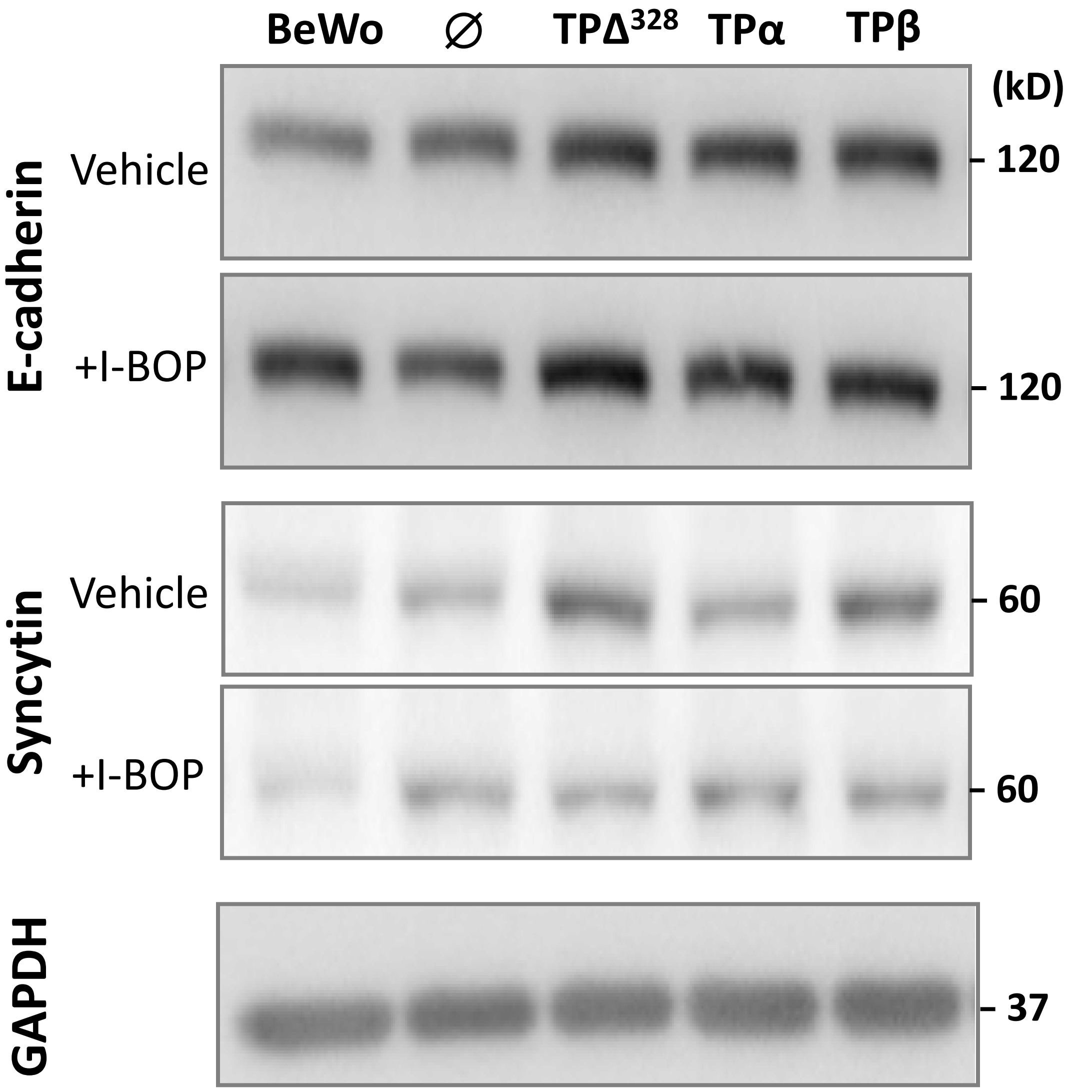

Supplementary Figure 3. TP activation under basal conditions did not alter trophoblast differentiation. Transfected BeWo cells were incubated for 72 hours with 200 nM I-BOP or vehicle control and changes in syncytialisation examined by immunoblotting for E-cadherin and syncytin. I-BOP stimulation (200 nM) did not change the expression of either differentiation marker in any of the TP expressing cell lines examined. GAPDH was used to control for protein loading and protein bands were quantified using densitometry. Blots are representative of 3 independent experiments.

Supplementary Figure 4

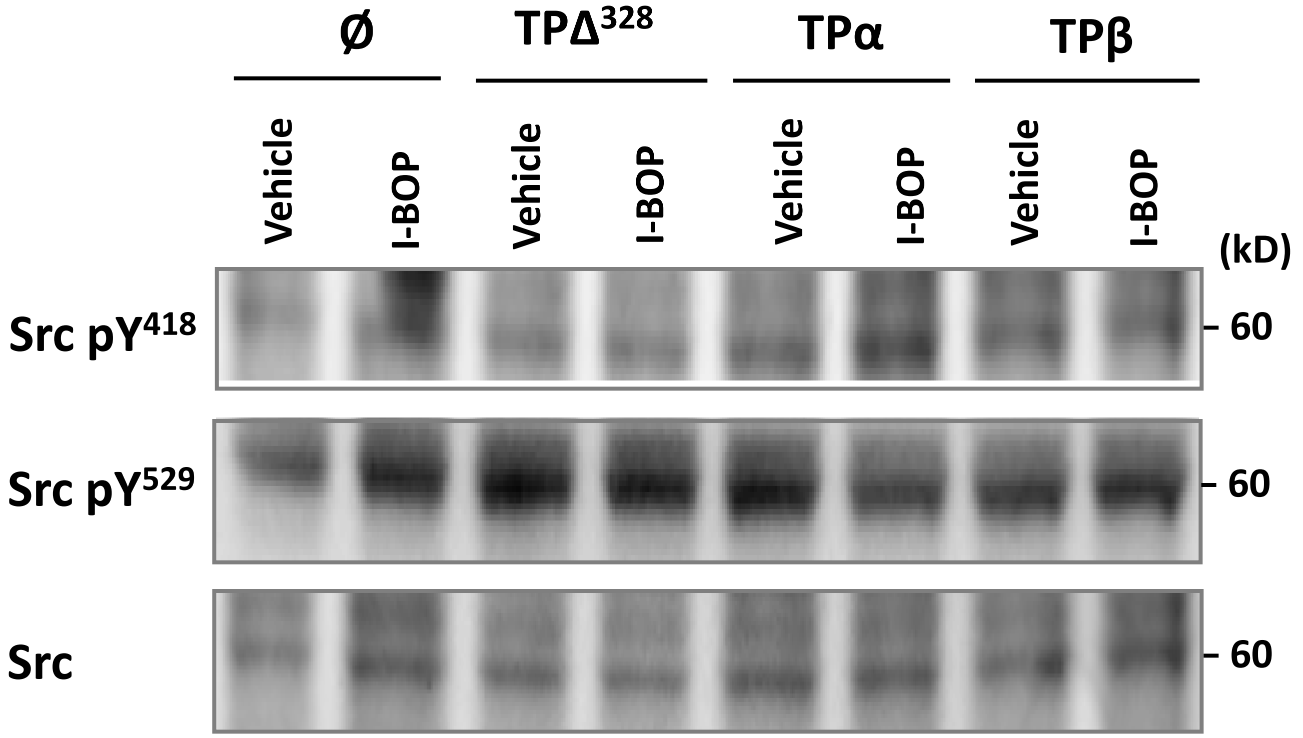

Supplementary Figure 4: Src activation was not altered by TPβ activation in JEG-3 cells. Immunoblotting of JEG-3 cells transfected with empty vector (∅), $TP\Delta^{328}$, TPα and TPβ was used to examine Src activation, which is a balance between phosphorylation at residues Y^{416} and Y^{527}. Blotting for the non-phosphorylated epitope in Src was used to control for protein loading and protein bands were quantified using densitometry. Blots are representative of 4 independent experiments.
